# Low-biomass pyruvate production with engineered *Vibrio natriegens* is accompanied by parapyruvate formation

**DOI:** 10.1186/s12934-025-02693-1

**Published:** 2025-03-28

**Authors:** Maurice Hädrich, Clarissa Scheuchenegger, Sören-Tobias Vital, Christoph Gunkel, Susanne Müller, Josef Hoff, Jennifer Borger, Erich Glawischnig, Felix Thoma, Bastian Blombach

**Affiliations:** 1 Microbial Biotechnology, Campus Straubing for Biotechnology and Sustainability, Technical University of Munich, Straubing, Germany; 2 SynBiofoundry@TUM, Technical University of Munich, Straubing, Germany; 3 Munich Institute of Integrated Materials, Energy and Process Engineering, Technical University of Munich, Garching, Germany

**Keywords:** *Vibrio natriegens*, Pyruvate, Parapyruvate, 4-hydroxy-4-methyl-2-oxoglutarate, Catalase, KatG, LigK, HMG/CHA aldolase, *Corynebacterium glutamicum*

## Abstract

**Background:**

Pyruvate is a precursor for various compounds in the chemical, drug, and food industries and is therefore an attractive target molecule for microbial production processes. The fast-growing bacterium *Vibrio natriegens* excels with its specific substrate uptake rate as an unconventional chassis for industrial biotechnology. Here, we aim to exploit the traits of *V. natriegens* for pyruvate production in fermentations with low biomass concentrations.

**Results:**

We inactivated the pyruvate dehydrogenase complex in *V. natriegens* Δ*vnp12*, which harbors deletions of the prophage regions *vnp12*. The resulting strain *V. natriegens* Δ*vnp12* Δ*aceE* was unable to grow in minimal medium with glucose unless supplemented with acetate. In shaking flasks, the strain showed a growth rate of 1.16 ± 0.03 h^− 1^ and produced 4.0 ± 0.3 g_Pyr_ L^− 1^ within 5 h. We optimized the parameters in an aerobic fermentation process and applied a constant maintenance feed of 0.24 g_Ac_ h^− 1^ which resulted in a maximal biomass concentration of only 6.6 ± 0.4 g_CDW_ L^− 1^ and yielded highly active resting cells with a glucose uptake rate (q_S_) of 3.5 ± 0.2 g_Glc_ g_CDW_^−1^ h^− 1^. *V. natriegens* Δ*vnp12* Δ*aceE* produced 41.0 ± 1.8 g_Pyr_ L^− 1^ with a volumetric productivity of 4.1 ± 0.2 g_Pyr_ L^− 1^ h^− 1^. Carbon balancing disclosed a gap of 30%, which we identified partly as parapyruvate. Deletion of *ligK* encoding the HMG/CHA aldolase in *V. natriegens* Δ*vnp12* Δ*aceE* did not impact biomass formation but plasmid-based overexpression of *ligK* negatively affected growth and led to a 3-fold higher parapyruvate concentration in the culture broth. Notably, we also identified parapyruvate in supernatants of a pyruvate-producing *Corynebacterium glutamicum* strain. Cell-free bioreactor experiments mimicking the biological process also resulted in parapyruvate formation, pointing to a chemical reaction contributing to its synthesis.

**Conclusions:**

We engineered metabolically highly active resting cells of *V. natriegens* producing pyruvate with high productivity at a low biomass concentration. However, we also found that pyruvate production is accompanied by parapyruvate formation in *V. natriegens* as well as in a pyruvate producing *C. glutamicum* strain. Parapyruvate formation seems to be a result of chemical pyruvate conversion and might be supported biochemically by an aldolase reaction.

**Supplementary Information:**

The online version contains supplementary material available at 10.1186/s12934-025-02693-1.

## Introduction

Pyruvate, in its protonated form called pyruvic acid, is a key intermediate in the central carbon metabolism and serves as precursor for various catabolic and anabolic routes such as the tricarboxylic acid (TCA) cycle or the biosynthesis of branched-chain amino acids. The three-carbon carboxylic acid has industrial relevance and is a precursor for various compounds, such as chemicals, fuels, pharmaceuticals, and polymers [1–3]. Examples of pyruvate-derived molecules which have been produced by microbial fermentation include lactate [4], butanol [5], isobutanol [6], 2,3-butandiol [7], alanine [8], l-valine [9], *N*-acetylneuraminic acid [10], and l-DOPA [11]. Additionally, pyruvate was identified as one of the most relevant precursors for a variety of non-native commercial products in *Escherichia coli* [12]. Pyruvate is also used as a dietary supplement due to its neuroprotective effects [13, 14] and might have beneficial effects on exercise performance [14, 15]. Pyruvate is produced predominantly by chemical processes such as dehydration and decarboxylation of tartaric acid [16]. Chemical pyruvate production suffers from the associated costs [17] and is ecologically questionable, as substrates are partially fossil-resource-derived. Therefore, extensive research for biotechnological pyruvate production from renewable resources has been carried out and several microorganisms such as *E. coli* [18], *Corynebacterium glutamicum* [19], *Lactococcus lactis* [20], as well as yeasts such as *Saccharomyces cerevisiae* [21], *Yarrowia lipolytica* [22], *Candida glabrata* [23], and engineered derivatives have been exploited for this purpose.

Under aerobic conditions, a major fraction of the intracellular pyruvate pool is utilized for the synthesis of acetyl-CoA. This oxidative decarboxylation is catalyzed by the pyruvate dehydrogenase complex (PDHC) which consists of three subunits [24]. Consequently, several metabolic engineering approaches have focused on abolishing or reducing the flux towards acetyl-CoA. This flux reduction was achieved by deletion of the *aceE* gene encoding the E1 subunit of the PDHC [25–28], silencing *aceE* expression by antisense RNA [29] or CRISPRi [30], enzyme engineering [31], promoter modification [32], and implementing cofactor auxotrophies [18, 22, 23]. Further strain engineering strategies aim to reduce by-product formation by deleting competing pathways [1–3]. Depending on the host organism, targets for gene deletion include for example the lactate dehydrogenase [19, 27, 28, 33], pyruvate oxidase [26–28], pyruvate decarboxylase [21], phosphoenolpyruvate synthase [27, 28, 33], and alanine aminotransferase [19]. Another way to improve pyruvate production is an increased glycolytic flux. Deletion of the F_1_-ATPase increased the glycolytic flux in *E. coli* [28, 34] and an ATPase inhibitor increased pyruvate yield in *C. glabrata* [35].

Parapyruvate, or 4-hydroxy-4-methyl-2-oxoglutarate (HMG), is a six-carbon dicarboxylic acid formed from two pyruvate molecules via aldol condensation [36]. It is commonly found as impurity in pyruvate supplements [37, 38]. In vitro, parapyruvate can be derived from pyruvate by alkaline treatments [38, 39]. Enzymatic degradation of parapyruvate to pyruvate was demonstrated by the aldolase LigK [40, 41]. In vivo, LigK is part of the protocatechuate (PCA) 4,5-cleavage pathway, catalyzing the last step from 4-carboxy-4-hydroxy-2-oxoadipate (CHA) to pyruvate and oxaloacetate [42]. However, in vivo parapyruvate formation by LigK has not been described as far as we know.

Biotechnological processes rely on the formation and maintenance of the biocatalyst, which may utilize a significant portion of the supplied carbon source and therefore reduce the achievable product yield. Moreover, high biomass concentrations may negatively impact downstream processing and separating biomass from the product and disposal of it are additional costs to consider [43–45]. Therefore, lowering biomass concentrations could be an option to reduce the overall process costs. Still, in order to achieve economic viability, key performance indicators (KPIs) of the fermentation, such as titer, productivity, and product yield, need to be high [46]. To maximize the KPIs with low biomass concentration, metabolically highly active cells are necessary, which is inherently linked to the substrate uptake rate of the biocatalyst. Therefore, a suitable candidate for this approach is *V. natriegens*, a non-pathogenic marine bacterium known for its high growth rate that can reach values of about 1.9 h^− 1^ in minimal medium with glucose as substrate [47]. The biomass-specific glucose uptake rate of *V. natriegens* during exponential aerobic growth is 3.9 g_Glc_ g_X_^−1^ h^− 1^, which is twice as high compared to established bacteria such as *E. coli* and *Bacillus subtilis* [48]. *V. natriegens* is a recently emerging unconventional host for biotechnological applications with rising research interest (reviewed in [49–51]). Plentiful genetic engineering tools have already been established and applied to *V. natriegens*, and the pool of parts for synthetic biology is steadily increasing, enabling rapid metabolic engineering of this bacterium [52, 53]. Previously, *V. natriegens* was engineered for pyruvate production [33] and pyruvate-derived products, such as 2,3-butanediol [54, 55], succinate [56, 57], alanine [48], and l-DOPA [58].

In this study, we explore the possibility for pyruvate production with *V. natriegens* in a low-biomass setup through metabolic engineering and process development. Furthermore, we highlight the formation of parapyruvate during pyruvate production and investigate the involvement of LigK in vivo, as well as chemical formation of parapyruvate.

## Results

### Pyruvate production in a low-biomass process

**Fig. 1:**
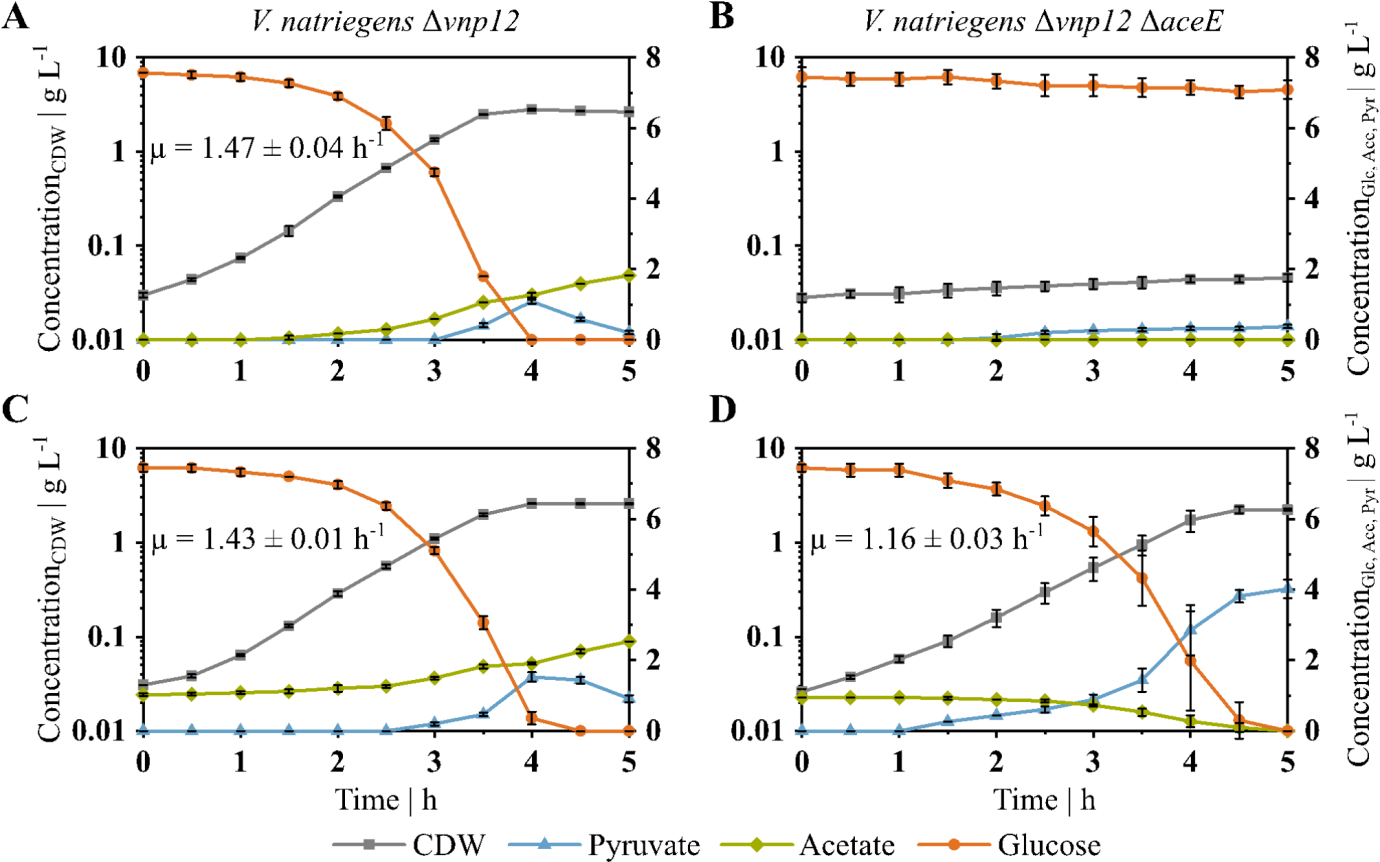
Growth characteristics of *V. natriegens* Δ*vnp12* and *V. natriegens* Δ*vnp12* Δ*aceE*. Plotted are the cell dry weight (CDW, grey, square), pyruvate (Pyr, blue, triangle), acetate (Ace, green, diamond) and glucose (Glc, orange, circle) concentrations over time. *V. natriegens* Δ*vnp12* (**A** and **C**) and *V. natriegens* Δ*vnp12* Δ*aceE* (**B** and **D**) were cultivated in 50 mL minimal medium, either with 7.5 g glucose L^-1^ as sole carbon source (**A** and **B**) or additionally with 1 g acetate L^-1^ (**C** and **D**). Strains were incubated in baffled shaking flasks at 37 °C. Data shown are means and standard deviations of independent triplicates

In a previous work, it was shown that *V. natriegens* harbors two prophage regions. Deletion of these regions led to a more stable and stress resistant growth behavior. Therefore, this prophage-free strain *V. natriegens* Δ*vnp12* was used as base for further strain engineering [59]. To disrupt the metabolic flux from pyruvate to acetyl-CoA, the *aceE* gene (PN96_01335) was deleted. Then, we cultivated the resulting strain *V. natriegens* Δ*vnp12* Δ*aceE* in VN minimal medium and characterized growth, substrate consumption and product formation (see Fig. 1). Deletion of the *aceE* gene caused the strain to lose the ability to grow on glucose as sole carbon and energy source (see Fig. 1A, B). Additional supplementation of acetate to the medium restored the growth of *V. natriegens* Δ*vnp12* Δ*aceE*, resulting in a growth rate (µ) of 1.16 ± 0.03 h^− 1^ compared to 1.43 ± 0.01 h^− 1^ of *V. natriegens* Δ*vnp12* under the same conditions (see Fig. 1C, D). While *V. natriegens* Δ*vnp12* accumulated acetate over time, the PDHC-deficient strain consumed the acetate simultaneously with the glucose. This strain’s growth was arrested once all acetate was taken up. *V. natriegens* Δ*vnp12* Δ*aceE* produced 4.0 ± 0.3 g_Pyr_ L^− 1^ after 5 h in shaking flasks, which corresponds to a product yield (Y_P/S_) of 0.54 ± 0.03 g_Pyr_ g_Glc_^−1^ (see Fig. 1D). Accordingly, analysis of intracellular metabolites showed that *V. natriegens* Δ*vnp12* Δ*aceE* exhibits a 6-fold higher concentration of pyruvate in the exponential growth phase compared to *V. natriegens* Δ*vnp12*.

**Fig. 2:**
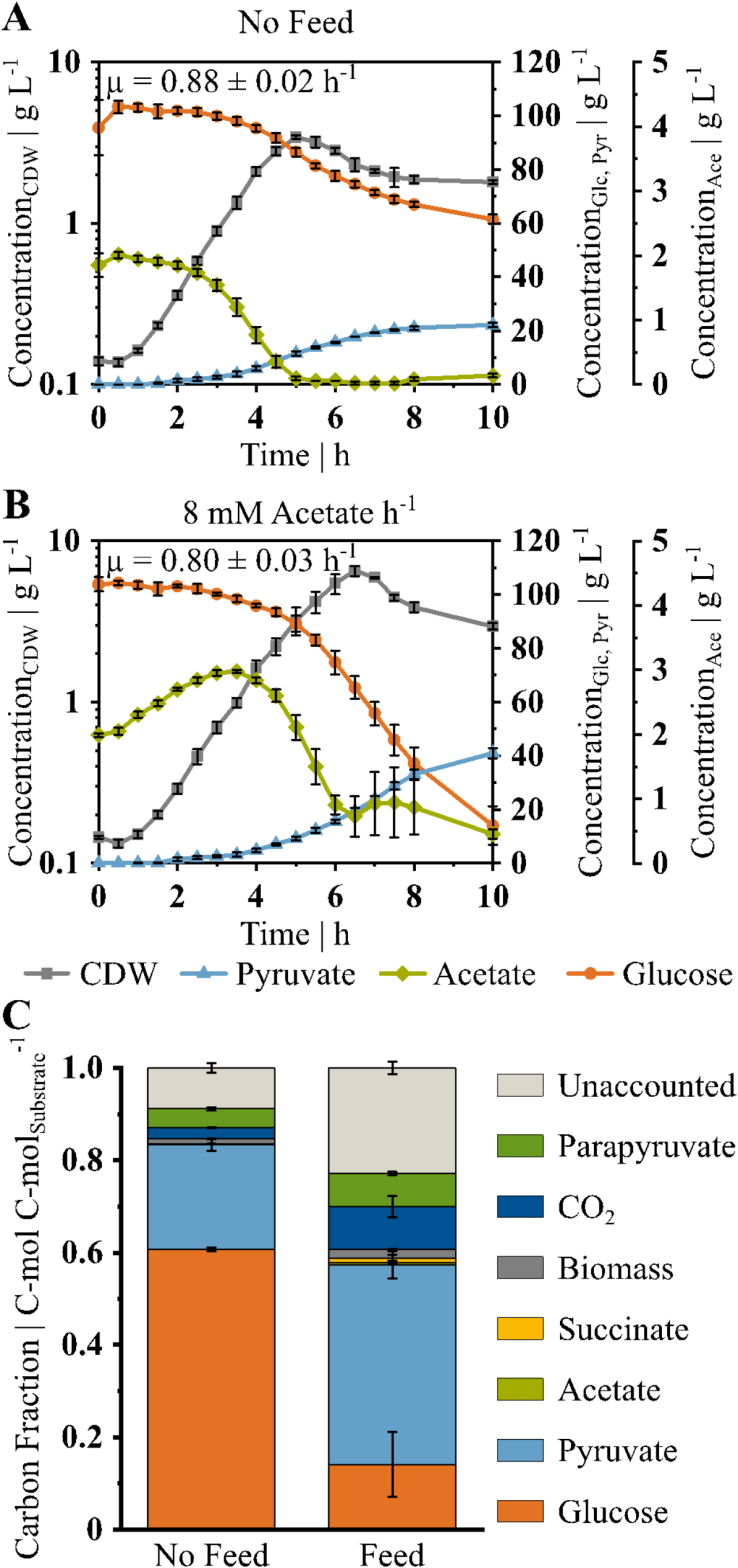
Pyruvate production in batch fermentations with *V. natriegens* Δ*vnp12* Δ*aceE*. Plotted are the cell dry weight (CDW, grey, square), pyruvate (Pyr, blue, triangle), acetate (Ace, green, diamond) and glucose (Glc, orange, circle) concentrations over time. Fermentations were performed in 500 mL minimal medium at 37 °C with (**A**) 100 g glucose L^-1^ and 2 g acetate L^-1^ as initial substrates and (**B**) using an additional feed of 0.24 g_Ac_ h^-1^. (**C**) Carbon distribution after 10 h in C-mol of found compound per C-mol of overall substrate for both bioreactor setups. Data shown are means and standard deviations of independent triplicates

*V. natriegens* Δ*vnp12* Δ*aceE* was then cultivated in a controlled bioreactor setup in 500 mL minimal medium with an initial substrate concentration of 100 g glucose L^− 1^. Biomass formation was limited by addition of 2 g acetate L^− 1^ to the medium. Upon depletion of acetate after 5 h, the strain reached a maximum biomass concentration (c_CDW_) of 3.4 ± 0.1 g_CDW_ L^− 1^ and showed a µ of 0.88 ± 0.02 h^− 1^ in the exponential growth phase (see Fig. 2A; Table 1). Glucose consumption and pyruvate production continued in the stationary phase to a titer of 22.2 ± 0.9 g_Pyr_ L^− 1^ after 10 h. The q_S_ decreased from 3.2 ± 0.3 g_Glc_ g_CDW_^−1^ h^− 1^ in the exponential phase to 1.9 ± 0.1 g_Glc_ g_CDW_^−1^ h^− 1^ in the stationary phase.

To enhance glucose utilization in the stationary phase, an acetate feed was applied (Fig. 2B). Considering a maintenance requirement of 0.063 g_Glc_ g_CDW_^−1^ h^− 1^ [60], we applied a constant feed of 0.24 g_Ac_ h^− 1^ from the start of the process. The acetate feed prolonged the growth phase with a µ of 0.80 ± 0.03 h^− 1^ to reach a peak biomass of 6.6 ± 0.4 g_CDW_ L^− 1^. Moreover, the applied feed maintained the q_S_ of the exponential phase of 3.2 ± 0.2 g_Glc_ g_CDW_^−1^ h^− 1^ in the stationary phase with 3.5 ± 0.2 g_Glc_ g_CDW_^−1^ h^− 1^ (see Table 1). Unlike the initial process, the acetate was not completely depleted from the medium when cell growth stopped and remained at a steady level. The highest pyruvate titer of 41.0 ± 1.8 g_Pyr_ L^− 1^ of the process was reached after 10 h with an Y_P/S_ of 0.51 ± 0.02 g_Pyr_ g_Glc_^−1^ and a volumetric productivity of 4.1 ± 0.2 g_Pyr_ L^− 1^ h^− 1^.

Analyzing the carbon balance after 10 h of cultivation revealed a gap of unaccounted carbon (see Fig. 2C). The amount increased with the feed applied to the process from about 13–30% and signals a so far unknown carbon sink. One additional compound that could be found in HPLC analysis of the supernatants was parapyruvate. The presence of parapyruvate in the sample was further verified by liquid chromatography–coupled quadrupole time-of-flight mass spectrometry (LC/Q-TOF-MS). For the reactor sample, at 12.4 min an m/z peak of 175.0251 was detected, corresponding to an C_6_H_7_O_6_^−^ ion (∂ 1.37 ppm), which was confirmed to represent parapyruvate by the purchased authentic standard (Rt 12.4 min, m/z of 175.0250, ∂ 0.94 ppm). However, absolute quantification was difficult as purchased standards showed additional peaks beside the main parapyruvate peak in the HPLC analysis (see Figure S1). Based on the theoretical concentration calculated with the area of the compound’s main peak, we estimated a concentration of 7 g parapyruvate L^− 1^ after 10 h in the process with acetate feed. This amount represents 24% of the previous unaccounted carbon fraction and about 17% of the total pyruvate yield.

**Table 1:** KPIs of *V. natriegens* Δ*vnp12* Δ*aceE* in fermentations without and with acetate feed (see also Fig. 2)

KPI	Without Feed	Acetate Feed
c_Pyr, max_| g_Pyr_ L^− 1^	22.2 ± 1.0	41.0 ± 1.8
Q_P_| g_Pyr_ L^− 1^ h^− 1^	2.2 ± 0.1	4.1 ± 0.2
q_S, exp_| g_Glc_ g_CDW_ h^− 1^	3.2 ± 0.3	3.2 ± 0.2
q_S, stat_| g_Glc_ g_CDW_ h^− 1^	1.9 ± 0.1	3.5 ± 0.2
Y_P/S_| g_Pyr_ g_Glc_^−1^	0.59 ± 0.03	0.51 ± 0.02
c_CDW, max_| g_CDW_ L^− 1^	3.4 ± 0.1	6.6 ± 0.4

*V. natriegens* is known to be sensitive to oxidative stress [50], and we speculated whether this susceptibility could negatively impact fermentations with resting cells as described above. Recently, the heterologous expression of a catalase improved the tolerance against cold-induced loss of viability as a result of oxidative stress [61]. Therefore, we integrated the *katG* gene, encoding a catalase from *E. coli*, into the well-tested *dns* locus [62, 63] in the genome of *V. natriegens* Δ*vnp12* Δ*aceE* to investigate whether *katG* expression would benefit fermentations with resting cells as alternative to the acetate feed. The resulting strain *V. natriegens* Δ*vnp12* Δ*aceE* Δ*dns*::*katG*_*Ec*_ was cultivated like the reference strain *V. natriegens* Δ*vnp12* Δ*aceE* in 500 mL minimal medium containing glucose and 2 g acetate L^− 1^ without additional feed. After 12 h cultivation time, no prolonged metabolic activity was observed and a large amount of leftover glucose remained again (see Figure S2). Noteworthy, compared to the parental strain, fermentations with *V. natriegens* Δ*vnp12* Δ*aceE* Δ*dns*::*katG*_*Ec*_ showed a significant 19% increase in the pyruvate titer from 24.5 ± 0.7 g_Pyr_ L^− 1^ to 30.3 ± 2.0 g_Pyr_ L^− 1^ (*p* < 0.05) and the pyruvate yield also increased by 30% from 0.45 ± 0.05 g_Pyr_ g_Glc_^−1^ to 0.64 ± 0.05 g_Pyr_ g_Glc_^−1^ (*p* < 0.05).

### The aldolase LigK can form parapyruvate in vivo

As enzymatic formation of parapyruvate was shown in vitro by the aldolase LigK [64], the genome of *V. natriegens* was screened for the PCA 4,5-cleavage pathway. This pathway consists of six enzymes [42] and all the required genes are present in the genome of *V. natriegens*, namely *ligA*, *ligB*, *ligI*, *ligU*, *ligJ* and *ligK* in a cluster (PN96_18525 to PN96_18550) and *ligC* close by (PN96_18475) (see Figure S3).

**Fig. 3:**
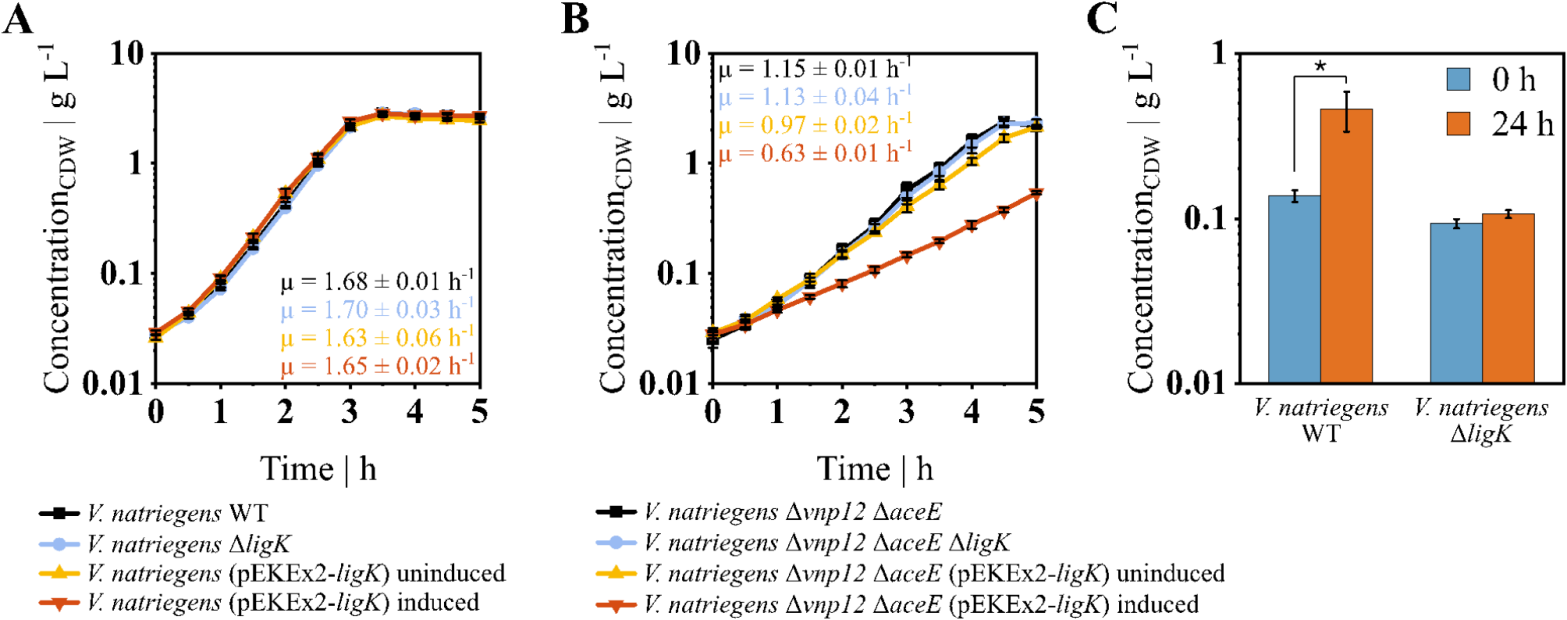
Growth of *V. natriegens* WT (**A**, black, square) and *V. natriegens* Δ*vnp12* Δ*aceE* (**B**, black, square) compared to respective *ligK* deletion strains (blue, circle), as well as both backgrounds with the overexpression plasmid pEKEx2-*ligK* uninduced (orange, triangle) and induced (red, inverted triangle). Cultivations were performed in baffled shaking flasks containing 50 mL minimal medium with 7.5 g glucose L^-1^ (**A** and **B**) and 1 g acetate L^-1^ (**B**) at 37 °C. (**C**) Growth of *V. natriegens* WT and *V. natriegens* Δ*ligK* with 10 mM PCA as only carbon and energy source in baffled shaking flasks containing 50 mL minimal medium at 37 °C. Data shown are means and standard deviations of independent triplicates. Asterisk indicates statistical significance of *p* < 0.05

To investigate in vivo parapyruvate production in *V. natriegens* via LigK, the corresponding gene was deleted or overexpressed with the plasmid pEKEx2 under the control of an inducible *tac* promoter, both in wild-type (WT) and *V. natriegens* Δ*vnp12* Δ*aceE*. In the WT background, no differences in growth behavior were observed in both the deletion and in the uninduced and induced overexpression strains (see Fig. 3A). Similarly, *V. natriegens* Δ*vnp12* Δ*aceE* Δ*ligK* did not exhibit a growth rate reduction with a µ of 1.13 ± 0.04 h^− 1^ compared to the parental strain, which had a µ of 1.15 ± 0.01 h^− 1^. However, unlike the WT, *V. natriegens* Δ*vnp12* Δ*aceE* showed a significant reduction of the growth rate upon overexpression of *ligK*, with a µ of 0.97 ± 0.02 h^− 1^ and 0.63 ± 0.01 h^− 1^ under uninduced and induced conditions, respectively (see Fig. 3B). To exclude the possibility of an unannotated aldolase being able to catalyze the last step of PCA degradation, *V. natriegens* WT and *V. natriegens* Δ*ligK* were grown on 10 mM PCA as sole carbon source. The WT strain showed biomass formation from PCA while the deletion strain did not, confirming the disruption of the pathway by inactivation of LigK (see Fig. 3C).

**Fig. 4:**
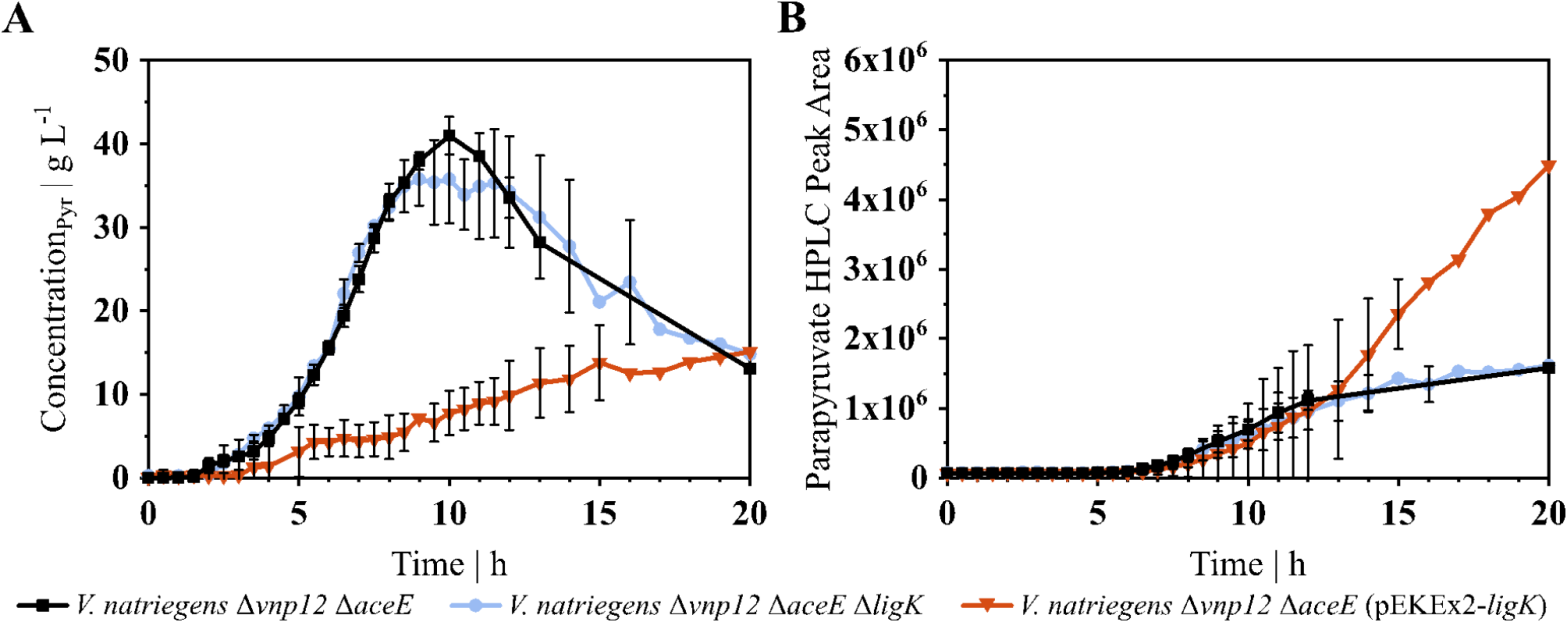
(**A**) Pyruvate and (**B**) parapyruvate formation in bioreactor cultivations of *V. natriegens* Δ*vnp12* Δ*aceE* (black, square), *V. natriegens* Δ*vnp12* Δ*aceE* Δ*ligK* (blue, circle) and induced *V. natriegens* Δ*vnp12* Δ*aceE* (pEKEx2-*ligK*) (red, inverted triangle). Strains were grown in 500 mL minimal medium at 37 °C with 100 g glucose L^-1^, 2 g acetate L^-1^ and a feed of 0.24 g_Ac_ h^-1^. Data points with error bars are means and standard deviations of independent triplicates. Data points without error bars represent means of duplicates

Then, *V. natriegens* Δ*vnp12* Δ*aceE* Δ*ligK* and *V. natriegens* Δ*vnp12* Δ*aceE* (pEKEx2-*ligK*) were cultured in a bioreactor to investigate the impact of LigK on pyruvate and parapyruvate formation. As observed in the shaking flasks, the *ligK* deletion mutant showed no differences compared to the parental strain *V. natriegens* Δ*vnp12* Δ*aceE*. In contrast, *V. natriegens* Δ*vnp12* Δ*aceE* (pEKEx2-*ligK*) with induced expression showed a strong shift in pyruvate and parapyruvate formation. Compared to *V. natriegens* Δ*vnp12* Δ*aceE*, the pyruvate titer of the strain harboring pEKEx2-*ligK* decreased by 78%, reaching 8.8 ± 1.4 g_Pyr_ L^− 1^ after 10 h process time (see Fig. 4A). Moreover, the parapyruvate amount of the overexpression strain was 3-fold higher (about 40 g L^− 1^) after 20 h compared to *V. natriegens* Δ*vnp12* Δ*aceE* and *V. natriegens* Δ*vnp12* Δ*aceE* Δ*ligK* (see Fig. 4B). This result indicates that LigK can catalyze the formation of parapyruvate under excess pyruvate conditions in vivo but also shows that another (bio)chemical mechanism is responsible for parapyruvate production in the tested strains (see Fig. 4).

To determine whether parapyruvate is a specific trait of *V. natriegens*, we also analyzed *C. glutamicum*, which does not harbor LigK and the PCA 4,5-cleavage pathway. LC/Q-TOF-MS analysis identified parapyruvate in the supernatant of the pyruvate-producing strain *C. glutamicum* Δ*aceE* Δ*pqo* Δ*ldhA* ΔC–T *ilvN* Δ*alaT* Δ*avtA* [19] but not in the supernatant of *C. glutamicum* WT after 72 h of incubation (data not shown). This result indicates that pyruvate production might be generally accompanied by parapyruvate formation.

### Cell-free parapyruvate formation

**Fig. 5:**
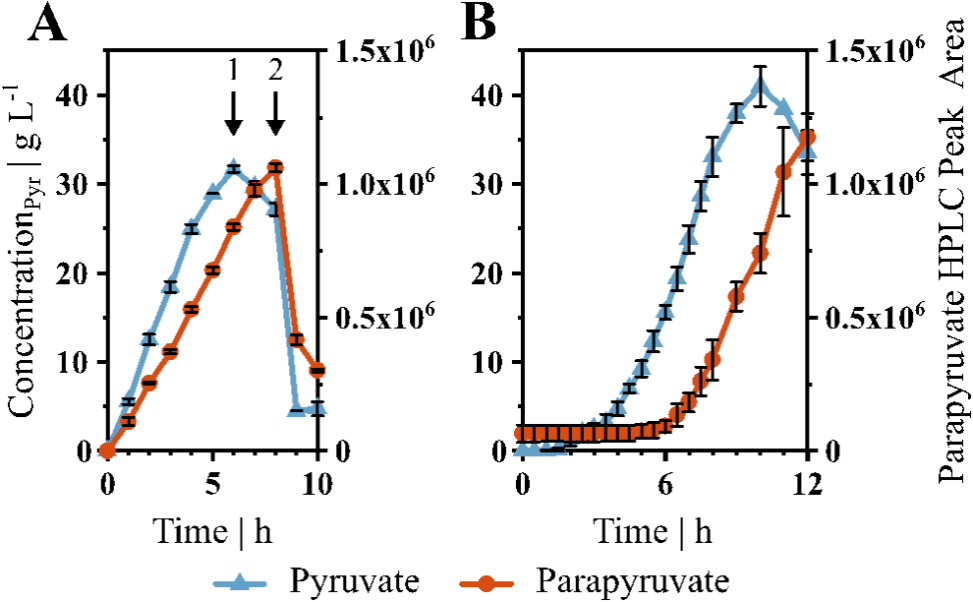
Pyruvate (Pyr, blue, triangle) and parapyruvate (red, circle) formation in the bioreactor over time. (**A**) Cell-free 500 mL minimal medium with a pyruvic acid feed of 7.5 g L^-1^ h^-1^. At 6 h (arrow 1), the feed was stopped and at 8 h (arrow 2), the pH was adjusted to 10. (**B**) *V. natriegens* Δ*vnp12* Δ*aceE* in 500 mL minimal medium at 37 °C with 100 g glucose L^-1^, 2 g acetate L^-1^ and a feed of 0.24 g_Ac_ h^-1^. Data shown are means and standard deviations of independent triplicates

Since the deletion of *ligK* did not reduce the parapyruvate formation, we explored other possibilities for its formation in the bioreactor. Parapyruvate is known to be formed chemically under alkaline conditions in vitro [38, 39]. In all bioreactor experiments, the pH was titrated to 7.5 with 25% ammonia solution to counteract the decrease in pH as a result of pyruvate production. The droplet-based addition might create micro-environments with high alkaline pH conditions, fostering parapyruvate formation. Therefore, we performed cell-free bioreactor experiments mimicking the microbial production process. A constant pyruvic acid feed was applied that acidified the medium demanding for pH control by titration with 25% ammonia solution. Pyruvate and parapyruvate concentrations were measured over time and showed a similar trend in the cell-free environment (see Fig. 5A) and the production process (see Fig. 5B). After 6 h in the cell-free experiments, the feed was stopped, but stirring continued for two more hours (see Fig. 5A, arrow 1). In this time, the pyruvate concentration started to decrease, while the parapyruvate titer still increased. After 8 h, the pH of the reactor was adjusted to 10 (see Fig. 5A, arrow 2) which resulted in a steep decrease in both pyruvate and parapyruvate concentration.

## Discussion

In this study, we engineered *V. natriegens* for pyruvate production, which we coupled to a metabolic switch for controlling biomass formation. This aim was achieved by deleting the *aceE* gene encoding the E1 subunit of the PDHC. As a result, the cells were only able to grow in the presence of glucose as long as acetate was available, which fed the TCA cycle with acetyl-CoA. The parental strain *V. natriegens* Δ*vnp12* showed exponential growth in medium with glucose and acetate, but acetate was not metabolized in parallel (see Fig. 1C), indicating typical catabolite repression. Due to an overflow metabolism, *V. natriegens* Δ*vnp12* additionally produced acetate under unlimited growth in the shaking flasks (see Fig. 1C). In contrast, the PDHC-deficient strain *V. natriegens* Δ*vnp12* Δ*aceE* consumed glucose and acetate in parallel, showing that the requirement for acetyl-CoA overcomes the global regulation by catabolite repression. Similarly, in PDHC-negative *E. coli* and *Pseudomonas putida* catabolite repression is relieved [27, 65], and glucose and acetate are utilized simultaneously [27]. PDHC-deficient strains of *C. glutamicum*, *Salmonella enterica* serovar Typhimurium, and *Pseudomonas aeruginosa* also exhibit an acetate auxotrophic phenotype [25, 66–68].

This phenotype allowed us to control biomass formation of *V. natriegens* Δ*vnp12* Δ*aceE* by adjusting the amount of acetate in the medium enabling a zero-growth production phase [69]. However, after the depletion of acetate, glucose consumption decreased rapidly from a q_S_ of 3.2 ± 0.3 g_Glc_ g_CDW_^−1^ h^− 1^ in the exponential growth phase to 1.9 ± 0.1 g_Glc_ g_CDW_^−1^ h^− 1^ in the stationary phase accompanied by a stop of pyruvate production after 10 h (see Fig. 2A; Table 1). To prolong the production phase, we applied a constant and low acetate feed of 0.24 g_Ac_ h^− 1^ which is in the range of the maintenance requirements determined for growth on glucose [60]. Interestingly, the acetate feed stimulated glucose utilization, and *V. natriegens* Δ*vnp12* Δ*aceE* showed a q_S_ of 3.5 ± 0.2 g_Glc_ g_CDW_^−1^ h^− 1^ under non-growing conditions that equals the q_S_ of the exponential growth phase of this process (see Table 1). This high q_S_ is an excellent basis for engineering zero-growth production processes for pyruvate-derived products with high productivity. Accordingly, *V. natriegens* Δ*vnp12* Δ*aceE* achieved a volumetric productivity of 4.1 ± 0.2 g_Pyr_ L^− 1^ h^− 1^ in our setup. This productivity exceeds that of other pyruvate production processes with engineered *E. coli* [2, 3], *C. glutamicum* [19], yeast [2, 3] and a recently constructed *V. natriegens* strain with attenuated PDHC activity [33] (see Table 2).

**Table 2:** Selection of glucose-based pyruvate production processes by different microorganisms

Strain	Process	Titer g L^− 1^	Yield g_Pyr_ g_Glc_^−1^	Productivity g L^− 1^ h^− 1^	Reference
*V. natriegens* Δ*vnp12* Δ*aceE*	Batch	41.0	0.51	4.1	This study
*V. natriegens* Δ*VPN1* Δ*VPN2* Δ*pflB* Δ*lldh* Δ*dldh* Δ*pps1* Δ*pps2* P2-*aceE*^TTG^ P2-*ppc*^ATG^	Fed-batch	54.2	0.57	3.4	[33]
*E. coli* K-12 Hfr *zbi*::Tn*10 poxB1* Δ(*aceEF*) *rpsL pps-4 pfl-1 ldhA*::Kan	Fed-batch	62.0	0.54	1.8	[27]
*E. coli* K-12 Hfr *zbi*::Tn*10 poxB1* Δ(*aceEF*) *rpsL pps-4 pfl-1 ldhA*::Kan *arcA726*::FRT *atpFH*::Cam	Fed-batch	90.0	0.68	2.1	[28]
*C. glutamicum* Δ*aceE* Δ*pqo* Δ*ldhA* ΔC–T *ilvN* Δ*alaT* Δ*avtA*	Fed-batch	45.0	0.47	0.4	[19]
*Evolved S. cerevisiae* MATa*pdc1*(− *6*,−*2*)::*loxP pdc5*(− *6*,−*2*)::*loxP pdc6*(− *6*,−*2*)::*loxP ura3*-*52*	Fed-batch	135.0	0.54	1.4	[21]
*Evolved C. glabrata* CCTCC M202019	Batch	94.3	0.64	1.2	[70]
*C. glabrata* IFO 0005	Batch	57.0	0.57	1.0	[23]

We applied the constant acetate feed from the onset of the process. Although acetate was not fully depleted in the medium, the cells stopped growing after 6 h at a biomass concentration of 6.6 ± 0.4 g_CDW_ L^− 1^ (see Fig. 2B). This growth arrest is probably not a result of a nutrient limitation since the WT of *V. natriegens* reached higher biomass concentrations in the same medium (data not shown). Therefore, another unknown regulatory mechanism such as quorum sensing [57], or an inhibitory effect of parapyruvate, as discussed below, may account for this behavior. A similar growth arrest was observed with a PDHC-deficient *E.coli* [28] and relieved by addition of betaine as osmoprotectant. As marine organism, *V. natriegens* has a variety of osmotic stress response systems [50]. Therefore, future studies may investigate the impact of osmotic pressure on pyruvate production.

Under optimized conditions, *V. natriegens* Δ*vnp12* Δ*aceE* produced 41.0 ± 1.8 g_Pyr_ L^− 1^ within 10 h with a product yield of 0.51 ± 0.02 g_Pyr_ g_Glc_^−1^ (see Table 1). Compared to other glucose-based microbial production processes, this product yield is at the lower end of the spectrum with values of up to 0.87 g_Pyr_ g_Glc_^−1^ [2, 3] and might be improved by applying an additional nitrogen limitation as reported for a PDHC-deficient *E. coli* strain overproducing pyruvate [28]. In our study, the carbon balance indicated the presence of a significant portion of unaccounted carbon in the fermentations of *V. natriegens* Δ*vnp12* Δ*aceE*, with and without acetate feed (see Fig. 2C). We did not observe typical pyruvate-derived side products such as lactate, alanine or valine, but identified parapyruvate as major carbon sink during pyruvate production in the bioreactor which accounted for 7% of the overall carbon. It was shown that parapyruvate can disrupt mitochondrial activity by inhibiting the α-ketoglutarate dehydrogenase complex [37, 71, 72]. Parapyruvate formation in the bioreactor might also impact the metabolism of *V. natriegens*, and could reasonably explain the growth arrest in the presence of acetate (see Fig. 2B) or the decreasing biomass concentration in the stationary phase (see Fig. 2A, B). To reduce parapyruvate formation, the aldolase LigK was inactivated, as the enzyme was shown to catalyze parapyruvate degradation and synthesis in vitro [64]. The deletion of *ligK* in *V. natriegens* Δ*vnp12* Δ*aceE* did not affect biomass, pyruvate or parapyruvate formation. However, the overexpression of *ligK* had a negative impact on growth and increased parapyruvate concentrations in the supernatant (see Figs. 3 and 4). Therefore, it is likely that the LigK enzyme is not expressed in *V. natriegens* Δ*vnp12* Δ*aceE* under the applied process conditions. LigK is part of the PCA 4,5-cleavage pathway and transcription is activated in other organisms by the regulator LigR in the presence of PCA or gallate [73]. A LigR homolog (PN96_18485) is also present in the genome of *V. natriegens*. These findings indicate potential challenges to use microorganisms harboring the PCA 4,5-cleavage pathway for pyruvate production from lignin-derived aromatic compounds because of potential LigK expression and increased parapyruvate formation.

Enzymatic parapyruvate formation by an unknown aldolase cannot be ruled out completely, but no other enzymes were identified with similarity to LigK in *V. natriegens* using BLAST [74]. It is unlikely that there is another aldolase catalyzing parapyruvate formation, since no growth on PCA was observed with *V. natriegens* Δ*vnp12* Δ*aceE* Δ*ligK*. In addition, cell-free experiments showed that parapyruvate is formed chemically in the bioreactor. Pyruvate can be converted to parapyruvate under various conditions, including a basic environment [38, 39] or by various inorganic catalysts such as ammonia [75] and cations [76, 77]. Parapyruvate was also found in fermentations with *Alcanivorax borkumensis* in minimal medium using pyruvate as sole carbon source [78] and during chemical production of pyruvate from H_2_ and CO_2_ using Ni_3_Fe particles [79]. As we also found parapyruvate in *C. glutamicum* cultures producing pyruvate, it appears to be a general by-product in microbial fermentations with high pyruvate concentrations. Notably, although several microbial systems have been engineered for pyruvate production, no study has reached the maximal theoretical product yield [3] indicating a general unaccounted byproduct such as parapyruvate. Parapyruvate can polymerize [38] and lactonize into zymonic acid [75] and different tautomers and hydrates [80] further complicating precise quantification of all aldol condensation products of pyruvic acid present in fermentation samples. Accordingly, after increasing the pH in the cell-free experiments not only the pyruvate but also the parapyruvate concentration decreased rapidly (see Fig. 5A). Bioreactors can have spatial heterogeneities [81] and our setup includes titration by dropping 25% ammonia solution from the top into the culture broth. This procedure could potentially lead to short term microenvironments with higher pH, fostering the aforementioned effects. Possibly, parapyruvate could be broken down to pyruvate in downstream processing by applying heat [82] but it could also decarboxylate to methylsuccinate [36]. The feasibility of downstream processing requires further research, but still neglects negative effects of parapyruvate on the cells during the process, when it is formed. Therefore, product recovery strategies [83] or fermentations at lower pH might inherently be better suited for pyruvate production, such as yeast processes at pH 4.5–5.5 [21–23].

## Conclusion

In this study, we expand the current research on pyruvate production with *V. natriegens* highlighting the potential of low-biomass fermentations with this chassis due to its outstanding substrate uptake rates. Major byproduct formation of parapyruvate was discovered to take place during pyruvate production with *V. natriegens* and other bacteria. Overexpression of the HMG/CHA aldolase (LigK) increased parapyruvate production from pyruvate during bioreactor experiments. However, our results indicate that parapyruvate formation mainly occurs chemically in a pH-dependent fashion and parapyruvate and its derivatives might therefore be present in most biotechnological pyruvate processes.

## Materials and methods

### Microorganisms and cultivation conditions

The bacterial strains and plasmids used in this study are listed in Table 3. For cloning purposes, *E. coli* was cultivated in 2xYT [84] and *V. natriegens* in modified LBv2 medium [57]. Unless otherwise specified, strains were incubated at 37 °C, with liquid cultures shaken at 180 rpm (Ø 25 mm, Multitron^®^2; INFORS GmbH, Bottmingen, Switzerland). Solid media for plates were prepared by adding 15 g agar L^− 1^. Strains were stored at -80 °C as 30% (v v^− 1^) glycerol stocks from a grown liquid culture.

**Table 3:** Strains and plasmids used in this study

Strain or plasmid	Relevant characteristics	Source or reference
**Strains**		
*E. coli* DH5α	F^−^ ϕ80*lacZ*ΔM15 Δ(*lacZYAarg*F)U169 *endA1 recA1 hsdR17* (r_K_^−^ m_K_^+^) *supE44* λ^−^*thi-1 gyrA96 relA1 phoA*	[86]
*E. coli* S17-1 λ*pir*	F^−^*thi pro hsdR hsdM*^+^*recA* RP4-2-Tc::Mu- Km::Tn7 λ*pir* Tp^R^ Sm^R^	[87]
*V. natriegens*	Wild-type strain DSM 759 (ATCC 14048), referred to as WT	German Collection of Microorganisms and Cell Cultures
*V. natriegens* Δ*ligK*	Deletion of *ligK* (PN96_18530) in *V. natriegens* WT	This study
*V. natriegens* Δ*vnp12*	Deletions of VNP regions 1 (PN96_04290 to PN96_04520) and 2 (PN96_06880 to PN96_07090) in *V. natriegens* WT	[59]
*V. natriegens* Δ*vnp12* Δ*aceE*	Deletion of *aceE* (PN96_01335) in *V. natriegens* Δ*vnp12*	This study
*V. natriegens* Δ*vnp12* Δ*aceE* Δ*dns*::*katG*_*Ec*_-1xFLAG	Replacing *dns* locus (PN96_00865) in *V. natriegens* Δ*vnp12* Δ*aceE* with *katG* from *E. coli* DH5α combined with a 1xFLAG tag	This study
*V. natriegens* Δ*vnp12* Δ*aceE* Δ*ligK*	Deletion of *ligK* (PN96_18530) in *V. natriegens* Δ*vnp12* Δ*aceE*	This study
*C. glutamicum*	Wild-type strain ATCC 13,032	[88]
*C. glutamicum* Δ*aceE* Δ*pqo* Δ*ldhA* ΔC–T *ilvN* Δ*alaT* Δ*avtA*	Engineered ATCC 13,032 for the production of pyruvate	[19]
**Plasmids**		
pDM4	*oriV*_R6K_, *oriT*_RP4_, *sacB*, Cm^R^	[89]
pDM4-Δ*aceE*	pDM4 plasmid carrying 500 bp homologous sequences upstream and downstream of *aceE*	This study
pST_116	*ori*_ColE1_, *tfoX*, *cas9*, *acrIIA4*, *sfgfp*, gRNA scaffold, *tetR*, *lacI*, Cm^R^	[63]
pST_116-Δ*ligK*	pST_116 with *sfgfp* exchanged for gRNA spacer targeting *ligK*	This study
pST_116-Δ*dns*	pST_116 with *sfgfp* exchanged for gRNA spacer targeting *dns*	This study
pET-22b(+)	*ori*_pBR322_, Carb^R^	Novagen
pJH001	*ori*_ColE1_, Carb^R^, 3 kb upstream and downstream flanks of *dns*, *sfgfp*	This study
pJH001- *katG*_*Ec*_-1xFLAG	pJH001 with *sfgfp* exchanged for *katG* from *E. coli* DH5α combined with a 1xFLAG tag	This study
pEKEx2	*oriV*_pBL1(*C.g*.)_, *oriV*_ColE1(*E.c*)_, P_*tac*_, *lacI*^*q*^, Kan^R^	[90]
pEKEx2-*ligK*	pEKEx2 with *ligK* under P_*tac*_ control	This study

For cultivations, *V. natriegens* glycerol stocks were streaked out on LBv2 agar plates. Single colonies were incubated overnight in test tubes containing 5 mL LBv2. Precultures in 500 mL baffled shaking flasks containing 50 mL VN medium at pH 7.5 [48] supplemented with 7.5 g glucose L^− 1^, were inoculated with 500 µL of the overnight culture. Cells were harvested after 3 to 4 h and used to inoculate main cultures. Shaking flask main cultures were inoculated with an OD_600_ of 0.1 and contained VN medium with 7.5 g glucose L^− 1^. VN medium for cultivations with *aceE*-deficient *V. natriegens* strains were additionally supplemented with 1 g acetate L^− 1^. Growth experiments with PCA were prepared accordingly with 10 mM PCA as sole carbon source in the main culture. Reactor cultivations were performed in a DASGIP Parallel Bioreactor System (Eppendorf, Jülich, Germany) with a vessel volume of 2 L using 0.5 L VN medium without MOPS, supplemented with 100 g glucose L^− 1^ and 2 g acetate L^− 1^ and inoculated with a starting OD_600_ of 0.5. The pH was maintained at 7.5 using a two-sided pH regulation using 13.3 M NH_4_OH and 1.1 M H_3_PO_4_. If specified, a constant acetate feed of 0.24 g_Ac_ h^− 1^ was applied. Reactors were aerated with pressurized air at 1 vvm and the agitation rate was adjusted between 400 and 1500 rpm to keep the dissolved oxygen (DO) above 50%. Cell-free experiments were performed in the bioreactor using the same settings except neither biomass nor acetate feed was added, and instead, a pyruvic acid feed of 7.5 g L^− 1^ h^− 1^ was applied. Where appropriate, media were supplemented with kanamycin (*E. coli* 50 µg mL^− 1^; *V. natriegens* 200 µg mL^− 1^) and chloramphenicol (*E. coli* 15 µg mL^− 1^; *V. natriegens* 6 µg mL^− 1^). Gene expression was induced by adding 1 mM isopropyl-β-d-thiogalactopyranosid (IPTG).

*C. glutamicum* was streaked out on a 2xYT plate from glycerol cultures. Single colonies were used to inoculate 5 mL 2xYT with 5 g acetate L^− 1^ in test tubes and cultivated over the day at 30 °C and 120 rpm. Precultures in 500 mL baffled shaking flasks containing 50 mL 2xYT supplemented with 5 g acetate L^− 1^ were inoculated with the whole culture of the test tube and cultivated over night at 30 °C and 120 rpm. Main cultures at 30 °C and 120 rpm were inoculated to an OD_600_ of 1 in CGXII medium [85] containing 40 g glucose L^− 1^, 10 g acetate L^− 1^ and 2 mM alanine.

### Strain construction

Genome reference and gene identifiers are used from GenBank assembly accession GCA_001456255.1 [91]. Oligonucleotides (Table S1) were purchased from either Sigma-Aldrich Chemie GmbH (Steinheim, Germany) or Eurofins MWG Operon (Ebersberg, Germany). Enzymes were purchased from New England Biolabs GmbH (Frankfurt am Main, Germany) and handled according to the manufacturer’s recommendations. The kits NucleoSpin^®^ Microbial DNA, NucleoSpin^®^ Plasmid and NucleoSpin^®^ Gel and PCR Clean-up (Macherey-Nagel, Düren, Germany) were used for the isolation and purification of genomic DNA, plasmid DNA and PCR fragments according to the manufacturer’s protocols. Electro-competent *E. coli* and *V. natriegens* cells were prepared as described [61, 84]. Sequencing was performed by Microsynth Seqlab GmbH (Göttingen, Germany).

Construction of pDM4-Δ*aceE* (primers #1/#2, #3/#4 and #5/#6), pST_116-Δ*ligK* (primers #9/#10 and #13/#14), and pST_116-Δ*dns* (primers #11/#12 and #13/#14) and subsequent deletion of *aceE* (primers #7/#8) and *ligK* (primers #15/#16, #17/#18 and #19/#20) was performed as previously described [57]. For the *katG* integration, first, the vector pJH001 containing 3 kb sequences up- and downstream of *dns* gene flanking a dropout *sfgfp* was constructed. As parts for pJH001, ColE1 origin of replication and *sfgfp* were amplified from pST_116 using primer pairs #33/#34 and #29/#30, respectively. Carbenecillin resistance gene was amplified from pET-22b(+) using primers pairs #21/#22 and #23/#24, removing a BsaI recognition site by silent mutation. *dns* flanking sequences were amplified from *V. natriegens*’ genomic DNA with primer pair #31/#32 for the upstream region and primer pairs #25/#26 and #27/#28 for the downstream region, removing an Esp3I recognition site. All primers added Esp3I recognition sites with matching overhangs and the plasmid was cloned by Golden Gate assembly using Esp3I as described [92]. pJH001- *katG*_*Ec*_-1xFLAG was constructed by amplifying the backbone from pJH001 including the *sfgfp* promotor and terminator with primer pair #35/#36 and *katG* was amplified from *E. coli* DH5α using primer pair #37/#38 introducing a 1xFLAG tag. The plasmid was cloned by Golden Gate assembly as described before. The transfer DNA (tDNA) was amplified using primer pair #39/#40 und subsequent integration via NT-CRISPR was performed as described before [63]. Integration was verified using primer pair #41/#42. Functional catalase activity was confirmed by adding 30% H_2_O_2_ to cell material and checking for visible oxygen formation.

For the overexpression of *ligK*, plasmid pEKEx2 was linearized with BamHI and EcoRI. The coding sequence of *ligK* was amplified from *V. natriegens* genomic DNA using primer pair #43/#44. Then, the fragment was assembled with the linearized plasmid pEKEx2 by Gibson Assembly [93]. Correct assemblies were screened in transformed *E. coli* by colony PCR with primer pair #45/#46 and subsequentially confirmed via Sanger sequencing before being introduced into *V. natriegens* by electroporation.

### Analytical methods

The biomass concentration was monitored by measuring the optical density (OD) at 600 nm with a spectrophotometer (Ultrospec^®^ 10, Biochrom, Holliston, MA, USA). OD values were converted into biomass concentrations applying a correlation factor of 0.28 g_CDW_ L^− 1^ per OD. Growth rates were calculated by fitting a linear regression line to the exponential growth phase in a semi-logarithmic plot and maximizing the coefficient of determination (R^2^). Determination of sugars and organic acids was performed by high-performance liquid chromatography (HPLC) with an Agilent 1260 infinity II series device (Agilent Technologies, Waldbronn, Germany) using a Hi-Plex H column (7.7 × 300 mm, 8 μm) and Hi-Plex Hguard cartridge (3.0 × 5.0 mm, 8 μm) as previously described [94]. Parapyruvate was purchased from Sigma-Aldrich with a stated quality of 95-105% by titration with HClO_4_.

For extraction of intracellular metabolites, strains were grown to an OD_600_ of 5 in shaking flasks. Cells were harvested from 2 mL culture by centrifugation (30 s, 20000 × g, 4 °C). The supernatant was removed, and cells were washed with 1 mL ice-cold 0.9% NaCl. After another centrifugation (30 s, 20000 × g, 4 °C) the supernatant was discarded and the cells were quenched in liquid nitrogen. Subsequently, cells were thawed on ice and resuspended in 0.5 mL ice-cold methanol. The suspension was vortexed and frozen in liquid nitrogen, before being incubated at -20 °C to thaw, vortexed and frozen in liquid nitrogen again. This was repeated until the cells incubated four times at -20 °C. The suspension was then centrifuged (10 min, 20000 × g, 4 °C) and supernatant stored at -80 °C until further analysis.

Sample preparation for the LC/Q-TOF-MS was performed as described [95]. 20 µL sample, either extracts of intracellular metabolites or culture supernatant, was mixed with 1 µL 1 M ammonium acetate (pH 9.2), 4 µL α-aminobutyrate (internal standard) and 15 µL Milli-Q H_2_O by vortexing. Subsequently, 60 µL acetonitrile was added, samples were vortexed and chilled on ice for 10 min before being centrifuged (10 min, 20000 × g, 4 °C). 90 µL supernatant were transferred to glass vials for measurement.

LC/Q-TOF-MS analysis was performed as described before [96] using a 1290 series UHPLC and 6546 LC/Q-TOF (Agilent Technologies, Waldbronn, Germany) equipped with a InfinityLab Poroshell 120 HILIC-Z (150 × 2.1 mm, 2.7 μm particle size). As eluent A, 1 M ammonium acetate was diluted in Milli-Q H_2_O (pH 9.2) and mixed with acetonitrile 1:9 (v/v) to a final concentration of 10 mM. Eluent B was 1 M ammonium acetate diluted in Milli-Q H_2_O (pH 9.2) and mixed with acetonitrile 9:1 (v/v) to a final concentration of 10 mM. MassHunter LC/MS Data Acquisition (v10.1), Qualitative Analysis software (v10.0), Masshunter Profnder (v10.0) and Mass Profiler Professional from Agilent Technologies were used for data acquisition and evaluation, respectively. Identity of pyruvate (Rt = 5.6 min, m/z: 87.009) was confirmed with an authentic standard.

## Electronic supplementary material

Below is the link to the electronic supplementary material.

Supplementary Material 1

## Data Availability

No datasets were generated or analysed during the current study.
