# Supplementary material for: Low-biomass pyruvate production with engineered Vibrio natriegens is accompanied by parapyruvate formation
Source: Microb Cell Fact. 2025 Mar 28;24:73. doi: 10.1186/s12934-025-02693-1 (PMC11951559; doi:10.1186/s12934-025-02693-1)
Supplement: Supplementary file 1 — Supplementary Material 1 [file 12934_2025_2693_MOESM1_ESM.docx]

**Supplementary data**

**Low-biomass pyruvate production with engineered *Vibrio natriegens* is accompanied by parapyruvate formation**

**Maurice Hädrich, Clarissa Scheuchenegger, Sören-Tobias Vital, Christoph Gunkel, Susanne Müller, Josef Hoff, Jennifer Borger, Erich Glawischnig, Felix Thoma, Bastian Blombach**

# Content

1. Comparison of commercial parapyruvate and reactor samples on the HPLC.
2. Comparison of *V. natriegens* Δ*vnp12* Δ*aceE* and *V. natriegens* Δ*vnp12* Δ*aceE* Δ*dns*::*katG_Ec_* in the bioreactor without acetate feed*.*
3. Protocatechuate 4,5-cleavage pathway and respective gene identifiers in *V. natriegens*.
4. Oligonucleotides used in this study.

Figure S1: HPLC chromatogram of commercial parapyruvate (black) in comparison to a bioreactor sample (red).

Figure S2: Pyruvate production in batch fermentations with (A) *V. natriegens* Δ*vnp12* Δ*aceE* and (B) *V. natriegens* Δ*vnp12* Δ*aceE* Δ*dns*::*katG_Ec_*. Plotted are the cell dry weight (CDW, grey, square), pyruvate (Pyr, blue, triangle), acetate (Ace, green, diamond) and glucose (Glu, orange, circle) concentrations over time. Fermentations were perfomed at 37 °C with 100 g glucose L^-1^ and 2 g acetate L^-1^ as initial substrates. Data shown are means and standard deviations of independent triplicates.

Figure S3: Protocatechuate (PCA) 4,5-cleavage pathway to oxaloacetate and pyruvate. All catalyzing enzymes and their respective gene identifiers in *V. natriegens* are indicated. The reaction of 4-hydroxy-4-methyl-2-oxoglutarate (parapyruvate) to pyruvate, also catalyzed by LigK, is added in comparison (orange arrow).

## Oligonucleotides used in this study

Table S1: Oligonucleotides used in this study

| **#** | **Oligonucleotide name** | **Sequence (5’ 🡪 3’)** | **Purpose** |
| --- | --- | --- | --- |
| 1 | fw_aceE_upstr_pDM4 | AGTTGAAGTGATGAGATCTATCTAGAGATGGAAGGTATTGCAGC | Construction of pDM4-Δ*aceE* |
| 2 | rv_aceE_upstr | TATCTACCTTCTTAAGGCGATCTATCCTTCTG | Construction of pDM4-Δ*aceE* |
| 3 | fw_aceE_dwnstr | GAAGGATAGATCGCCTTAAGAAGGTAGATAAGAAATGGC | Construction of pDM4-Δ*aceE* |
| 4 | rv_aceE_dwnstr_pDM4 | ATATCAAGCTTATCGATACCGTCGACCTTTGATTTCTTTAAGCGTACC | Construction of pDM4-Δ*aceE* |
| 5 | fw_pDM4_seq | GACATGGAGCGGCACGC | Verification of pDM4-Δ*aceE* |
| 6 | rv_pDM4_seq | GCAACTGACTGAAATGCC | Verification of pDM4-Δ*aceE* |
| 7 | fw_aceE_seq | GGCGGTCGAGAAAGTAAG | Verification of *aceE* deletion |
| 8 | rv_aceE_seq | CTGCTGGAGCTGCTTCC | Verification of *aceE* deletion |
| 9 | fw_gRNA_ligK | GTCCGTGATGGTCGCTGATGAAGA | Construction of pST_116-Δ*ligK* |
| 10 | rv_gRNA_ligK | AAACTCTTCATCAGCGACCATCAC | Construction of pST_116-Δ*ligK* |
| 11 | fw_gRNA_dns | GTCCCAGCTGCAATGCTGGCAAAG | Construction of pST_116-Δ*dns* |
| 12 | rv_gRNA_dns | AAACCTTTGCCAGCATTGCAGCTG | Construction of pST_116-Δ*dns* |
| 13 | fw_pST116_seq | CGACCTCATACGAGAGATTAAG | Verification of pST_116 variants |
| 14 | rv_pST116_seq | GGATAAGGCGCAGCG | Verification of pST_116 variants |
| 15 | fw_ligK_upstr | ACTATACCACTACACCACCG | Construction of Δ*ligK* tDNA |
| 16 | rv_ligK_upstr | GGTTAGACATAATATTAATCCTTATGCCTTCAGACG | Construction of Δ*ligK* tDNA |
| 17 | fw_ligK_dwnstr | GGATTAATATTATGTCTAACCAAGCCCCT | Construction of Δ*ligK* tDNA |
| 18 | rv_ligK_dwnstr | CAGCTTGGACTTGGTTCT | Construction of Δ*ligK* tDNA |
| 19 | fw_ligK_seq | GACTTCATCGGCTGTAACC | Verification of *ligK* deletion |
| 20 | rv_ligK_seq | GTTACTTCAACACCGTCAATC | Verification of *ligK* deletion |
| 21 | fw_carb_to_ColE1 | AAAACGTCTCCTCACAAGATCCTTTGATCTTTTCTACGG | Construction of pJH001 |
| 22 | rv_carb_BsaI_removal | AAAACGTCTCCCCCGCGGTATCATTGCAGCAC | Construction of pJH001 |
| 23 | fw_carb_BsaI_removal | AAAACGTCTCCCGGGACCCACGCTCACCGG | Construction of pJH001 |
| 24 | rv_carb_to_upstr | AAAACGTCTCCCCAAAGGTGGCACTTTTCGGGG | Construction of pJH001 |
| 25 | fw_dns_dwnstr | AAAACGTCTCCTTGGCTTTAAAAGCCTTGAGCG | Construction of pJH001 |
| 26 | rv_dns_dwnstr_Esp3I_removal | AAACGTCTCCGAAACGCTTCTCGGTCCGC | Construction of pJH001 |
| 27 | fw_dns_dwnstr_Esp3I_removal | AAAACGTCTCCTTTCTTCAAATTGGTACTCGCGC | Construction of pJH001 |
| 28 | rv_dns_dwnstr_to_sfgfp | AAAACGTCTCCCTCCTCCTCACCAATCGCGAC | Construction of pJH001 |
| 29 | fw_sfgfp_to_dns_dwnstr | AAAACGTCTCCGGAGCGAGACCGAAAGTGAAACGTGATTTCATGC | Construction of pJH001 |
| 30 | rv_sfgfp_to_dns_upstr | AAAACGTCTCCAGCGTGAGACCTATAAACGCAGAAAGGCCCAC | Construction of pJH001 |
| 31 | fw_dns_upstr_to_sfgfp | AAAACGTCTCCCGCTAGTTAAAGTCTTTAAAAAGTATGACTTTATC | Construction of pJH001 |
| 32 | rv_dns_upstr_to_ColE1 | AAAACGTCTCCGTTCGTGAGACTTACGTTAAAACCGG | Construction of pJH001 |
| 33 | fw_ColEI_to_dns_upstr | AAAAAACGTCTCCGAACCGTAAAAAAGGCCGC | Construction of pJH001 |
| 34 | rv_ColEI_to_carb | AAAAACGTCTCCGTGAAGATCCTTTTTGATAATCTCATG | Construction of pJH001 |
| 35 | rv_sfgfp_promotor_to_katG | AAAACGTCTCCCATCTAGTATTTCTCCTCTTTCTCTAG | Construction of pJH001- *katG_Ec_*-1xFLAG |
| 36 | fw_sfgfp_terminator_to_katG | AAAACGTCTCCCCAGGCATCAAATAAAACGAAAG | Construction of pJH001- *katG_Ec_*-1xFLAG |
| 37 | fw_ katG_to_sfgfp_promotor | AAAACGTCTCCGATGAGCACGTCAGACGATATC | Construction of pJH001- *katG_Ec_*-1xFLAG |
| 38 | rv_ katG-1xFLAG_to_sfgfp_terminator | AAAACGTCTCCCTGGTTACTTGTCATCGTCGTCCTTGTAGTCCAGCAGGTCGAAACGGTC | Construction of pJH001- *katG_Ec_*-1xFLAG |
| 39 | fw_tDNA_katG | TTGGCTTTAAAAGCCTTGAGCG | Construction of *katG_Ec_*-1xFLAG tDNA |
| 40 | rv_tDNA_katG | GTGAGACTTACGTTAAAACCGG | Construction of *katG_Ec_*-1xFLAG tDNA |
| 41 | fw_dns_seq | GCTGCACGACATATCCC | Verification of *dns* integration |
| 42 | rv_dns_seq | ACCTTCCTTCATACGAAGTAC | Verification of *dns* integration |
| 43 | fw_ligK_pEKEx2 | GCCTGCAGGTCGACTCTAGAGGATCCATGCAAAACAATGTTGTAGTTCAG | Construction of pEKEx2-*ligK* |
| 44 | rv_ligK_pEKEx2 | CTGTAAAACGACGGCCAGTGAATTCTTAGACATATTTGAGCCCTTTTTC | Construction of pEKEx2-*ligK* |
| 45 | fw_pEKEx2_seq | GCACTCCCGTTCTGGATAATGTT | Verification of pEKEx2-*ligK* |
| 46 | rv_pEKEx2_seq | GCGTTCTGATTTAATCTGTATCAGGCTG | Verification of pEKEx2-*ligK* |
|  |  |  |  |
